# Supplementary material for: Anti-Inflammatory, Antioxidant, and Wound-Healing Properties of Cyanobacteria from Thermal Mud of Balaruc-Les-Bains, France: A Multi-Approach Study
Source: Biomolecules. 2020 Dec 29;11(1):28. doi: 10.3390/biom11010028 (PMC7824682; doi:10.3390/biom11010028)
Supplement: Supplementary file 1 [file biomolecules-11-00028-s001.zip › Demay et al._supp. data_Table S11.docx]

Article

Anti-inflammatory, antioxidant and wound healing properties of cyanobacteria from thermal mud (Balaruc-les-Bains, France): a multi-approach study

Justine Demay ^1,2^, Sébastien Halary ^1^, Adeline Knittel-Obrecht ^3,4^, Pascal Villa ^3,4^, Charlotte Duval ^1^, Sahima Hamlaoui ^1^, Théotime Roussel ^1^, Claude Yéprémian ^1^, Anita Reinhardt ^2^, Cécile Bernard ^1,^*, and Benjamin Marie ^1,^*

^1^ UMR7245 MCAM MNHN-CNRS, Muséum National d’Histoire Naturelle, CP 39, 12 rue Buffon, F-75231 Paris Cedex 05, France.; [justine.demay1@mnhn.fr](mailto:justine.demay1@mnhn.fr) (J.D.) ; [sebastien.halary@mnhn.fr](mailto:sebastien.halary@mnhn.fr) (S.H.) ; [charlotte.duval@mnhn.fr](mailto:charlotte.duval@mnhn.fr) (C.D.) ; [sahima.hamlaoui@mnhn.fr](mailto:sahima.hamlaoui@mnhn.fr) (Sa.H.) ; [theotime.roussel@mnhn.fr](mailto:theotime.roussel@mnhn.fr) (T.H.) ; [claude.yepremian@mnhn.fr](mailto:claude.yepremian@mnhn.fr) (C.Y.)

^2^ Thermes de Balaruc-Les-Bains, 1 rue du Mont Saint-Clair BP 45, 34540 Balaruc-Les-Bains, France.; [anita.reinhardt@thermesbalaruc.com](mailto:anita.reinhardt@thermesbalaruc.com) (A.R.)

^3^

CNRS, Université de Strasbourg, PCBIS Plate-forme de Chimie Biologique Intégrative de Strasbourg UMS

3286, F-67412 Illkirch, France; [aobrecht@unistra.fr](mailto:aobrecht@unistra.fr) (A.O) ; [pvilla@unistra.fr](mailto:pvilla@unistra.fr) (P.V.)

^4^ Labex MEDALIS, F-67000 Strasbourg, France

***** Correspondence: [cecile.bernard@mnhn.fr](mailto:cecile.bernard@mnhn.fr) - Tel.: +33 1 40 79 31 83 (C.B.) ; [bmarie@mnhn.fr](mailto:bmarie@mnhn.fr) – Tel. : +33 1 40 79 32 12 (B.M.)

Received: date; Accepted: date; Published: date

Supplementary data

Table S11. Cell viability bioassays were assessed on the three cell types used for other activity tests (RAW 264.7; HaCaT; PMBC). The reliable toxicity threshold is set at a 40% decrease in cell viability.

|  |  |  | *P. raciborskii*  PMC 877.14 | | *Laspinema* sp.  PMC 878.14 | | *M. vaginatus*  PMC 879.14 | | *L. martensiana*  PMC 880.14 | | *Nostoc* sp.  PMC 881.14 | | *Aliinostoc* sp.  PMC 882.14 | | *L. boryana*  PMC 883.14 | | *Calothrix* sp.  PMC 884.14 | | *P. couteii*  PMC 885.14 | | |
| --- | --- | --- | --- | --- | --- | --- | --- | --- | --- | --- | --- | --- | --- | --- | --- | --- | --- | --- | --- | --- | --- |
| Bioassay |  | µg.mL^-1^ | MeOH | H_2_O | MeOH | H_2_O | MeOH | H_2_O | MeOH | H_2_O | MeOH | H_2_O | MeOH | H_2_O | MeOH | H_2_O | MeOH | H_2_O | MeOH | H_2_O |  |
| Cell Viability | RAW 264.7 | 1 | - | | - | -31 +/-2% | - | | - | -33+/-1% | - | -38+/-1% | - | -32+/-2% | - | | - | | - | |  |
|  |  | 5 | - | | - | -32+/-2% | - | | - | -34+/-5% | -32+/-1% | -41+/-0% | - | -36+/-3% | - | | - | | - | |  |
|  |  | 10 | - | | - | -30+/-2% | - | | - | -28+/-6% | -35+/-1% | -37-/2% | - | | - | | - | | - | |  |
|  |  | 50 | - | | - | | - | | -28+/-2% | - | - | | -28+/-3% | - | - | | - | -32+/-1% | - | |  |
|  | HaCaT | 1 | - | | | | | | | | | | | | | | | | | |  |
|  |  | 5 | - | | | | | | | | | | | | | | | | | |  |
|  |  | 10 | - | | | | | | | | | | | | | | | | | |  |
|  |  | 50 | - | | | | | | | | | | | | | | | | | |  |
|  | PBMC | 1 | - | | | | | | | | | | | | | | | | | |  |
|  |  | 5 | - | | | | | | | | | | | | | | | | | |  |
|  |  | 10 | - | | | | | | | | | | | | | | | | | |  |
|  |  | 50 | - | | | | | | | | | | | | | | | | | |  |
