# Supplementary material for: Anti-Inflammatory, Antioxidant, and Wound-Healing Properties of Cyanobacteria from Thermal Mud of Balaruc-Les-Bains, France: A Multi-Approach Study
Source: Biomolecules. 2020 Dec 29;11(1):28. doi: 10.3390/biom11010028 (PMC7824682; doi:10.3390/biom11010028)
Supplement: Supplementary file 1 [file biomolecules-11-00028-s001.zip › Figure S1_Consensus phylogenetic tree based on 16S rRNA gene sequences.pdf]

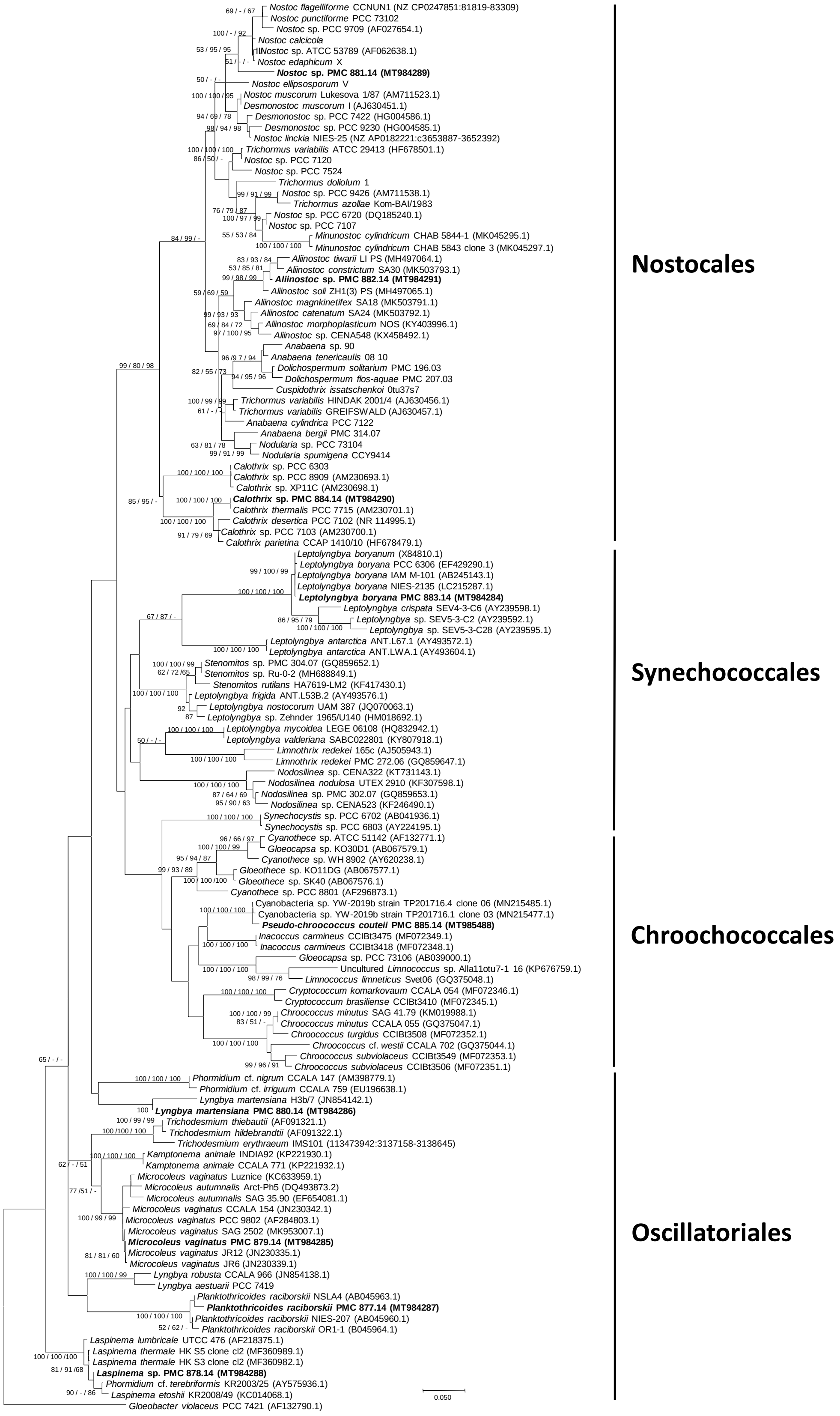

Figure S1. Consensus phylogenetic tree based on 16S rRNA gene sequences of representative cyanobacteria strains belonging to the orders Oscillatoriales, Nostocales, Chroococcales and Synechococcales, Balaruc-les-Bains strains (in bold) and one outgroup (*Gloeobacter violaceus*). Numbers above branches indicate bootstrap support (>50%) from 1000 replicates. Bootstrap values are given in the following order: neighbor joining/maximum likelihood/maximum parsimony.
