# Supplementary material for: Anti-Inflammatory, Antioxidant, and Wound-Healing Properties of Cyanobacteria from Thermal Mud of Balaruc-Les-Bains, France: A Multi-Approach Study
Source: Biomolecules. 2020 Dec 29;11(1):28. doi: 10.3390/biom11010028 (PMC7824682; doi:10.3390/biom11010028)
Supplement: Supplementary file 1 [file biomolecules-11-00028-s001.zip › Figure S2. Molecular network_coloured by extraction.pdf]

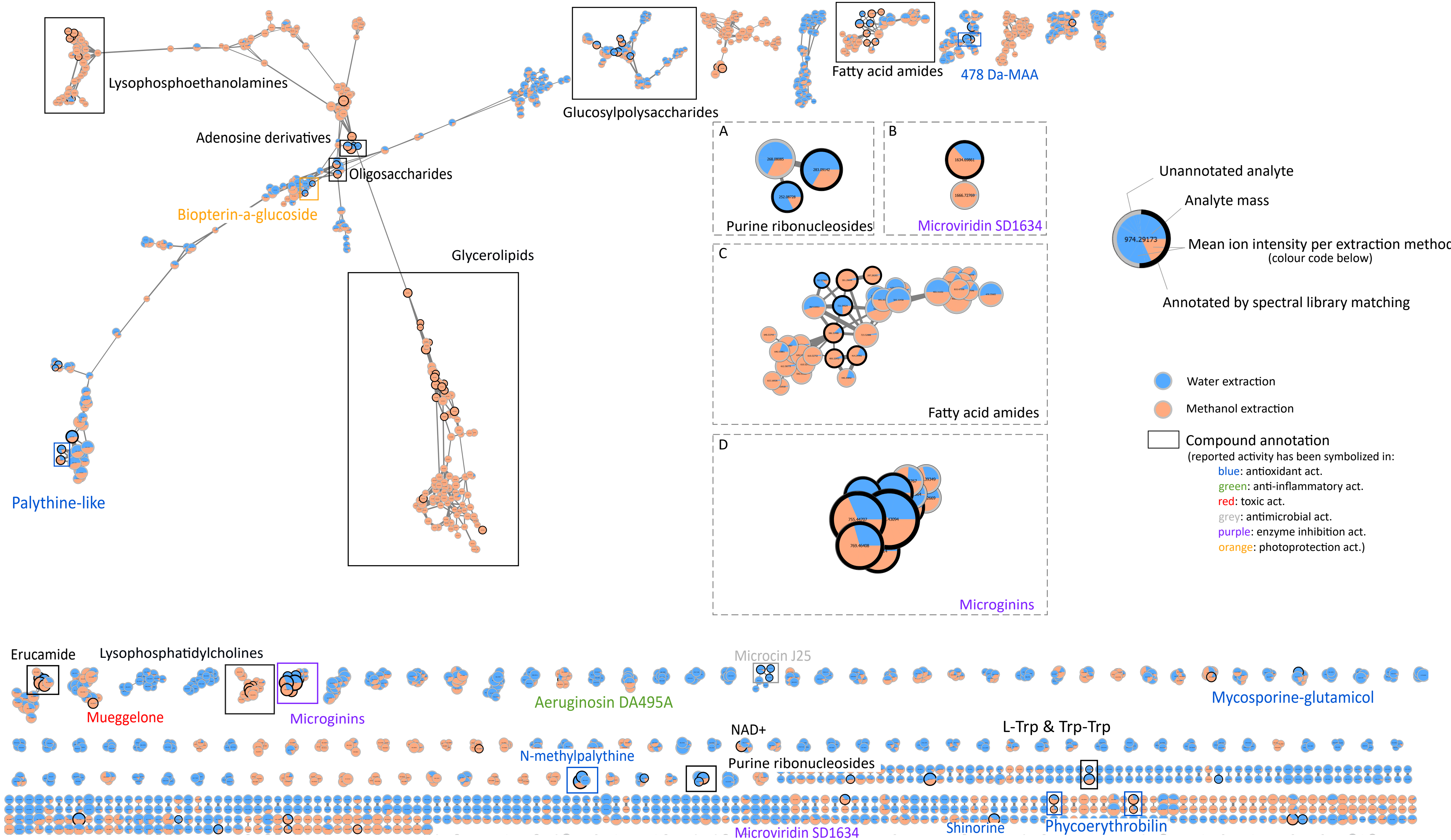

Figure S2. Molecular network of the metabolites produced by the nine cyanobacteria isolated from Balaruc thermal mud. Annotations obtained by untargeted analysis are figured inboxes. Nodes have been coloured according to the fraction of the extraction in which they were detected.
